# Innate lymphoid cells: a new key player in atopic dermatitis

**DOI:** 10.3389/fimmu.2023.1277120

**Published:** 2023-10-16

**Authors:** Haiping Jia, Huiying Wan, Dingding Zhang

**Affiliations:** ^1^ Institute of Basic Medicine and Forensic Medicine, North Sichuan Medical College, Nanchong, China; ^2^ Department of Dermatology, Sichuan Provincial People’s Hospital, University of Electronic Science and Technology of China, Chengdu, China; ^3^ Sichuan Provincial Key Laboratory for Genetic Disease, Sichuan Provincial People’s Hospital, University of Electronic Science and Technology of China, Chengdu, China

**Keywords:** innate lymphoid cells, atopic dermatitis, inflammatory response, pathophysiology, cytokines

## Abstract

Atopic dermatitis (AD) is a common allergic inflammatory skin condition mainly caused by gene variants, immune disorders, and environmental risk factors. The T helper (Th) 2 immune response mediated by interleukin (IL)-4/13 is generally believed to be central in the pathogenesis of AD. It has been shown that innate lymphoid cells (ILCs) play a major effector cell role in the immune response in tissue homeostasis and inflammation and fascinating details about the interaction between innate and adaptive immunity. Changes in ILCs may contribute to the onset and progression of AD, and ILC2s especially have gained much attention. However, the role of ILCs in AD still needs to be further elucidated. This review summarizes the role of ILCs in skin homeostasis and highlights the signaling pathways in which ILCs may be involved in AD, thus providing valuable insights into the behavior of ILCs in skin homeostasis and inflammation, as well as new approaches to treating AD.

## 1 Introduction

Atopic dermatitis (AD) is a chronic skin disease characterized by a massive infiltration of inflammatory cells, with intense pruritus, plasmacytic exudates, dry skin, and erythematous papules as the predominant clinical symptoms (1). The onset of AD is not limited by age or race. AD plays a significant role in the global burden of dermatologic diseases and has a detrimental impact on the quality of life of patients and their families. From 1990 to 2017, AD ranked 15th among all non-fatal diseases and the first among dermatological diseases in disability-adjusted life years (DALYs) (2). Traditionally, the pathogenesis of AD is highly complex, involving genetic predisposition, epidermal dysfunction, and T-cell-driven inflammation. The T helper (Th) 2 cells dominate the pathogenesis of AD by secreting pro-inflammatory cytokines such as interleukin (IL)-4 and IL-13. Dupilumab is a monoclonal antibody that selectively blocks IL-4 and IL-13 signaling and received the first global approval for AD treatment in March 2017, representing a major advance in treating patients with moderate-to-severe AD (3, 4). However, dupilumab is ineffective in some AD patients and might induce new regional dermatoses, ocular complications, alopecia, and other adverse effects (5). Although there is no accurate cure for AD, many novel and targeted therapies promise to slow the disease’s progression considerably, especially in patients with refractory AD. In recent years, the detection of innate lymphoid cells (ILCs) in the context of skin homeostasis and inflammation has gained increasing attention (6).

ILCs are a newly found lymphoid lineage component of the innate immune system that differentiates from common lymphoid progenitor cells (CLPs) (7, 8) and produce a range of cytokines associated with subsets of T helper cells (9). Furthermore, ILCs are characterized by the absence of antigen-specific receptors produced by genetic recombination (8), and their growth is typically dependent on the common gamma chain of the IL-2 receptor, Notch, and the transcription factor inhibitor of DNA binding 2 (ID2) (7). ILCs are crucial in generating immune responses, maintaining tissue integrity, and mediating inflammatory responses (8). Recent studies have demonstrated that the pathophysiology of AD is strongly connected to abnormal ILC activation (10, 11).

This review presents the involvement and function of ILCs in the skin, emphasizing the role of several subgroups of ILCs in the pathogenesis of AD, and further discusses the possible associated signaling pathways. The aim is to shed new light on the molecular mechanisms of AD and imply the potential value of targeting ILCs for therapy.

## 2 The subsets of the ILC family

**Figure 1:**
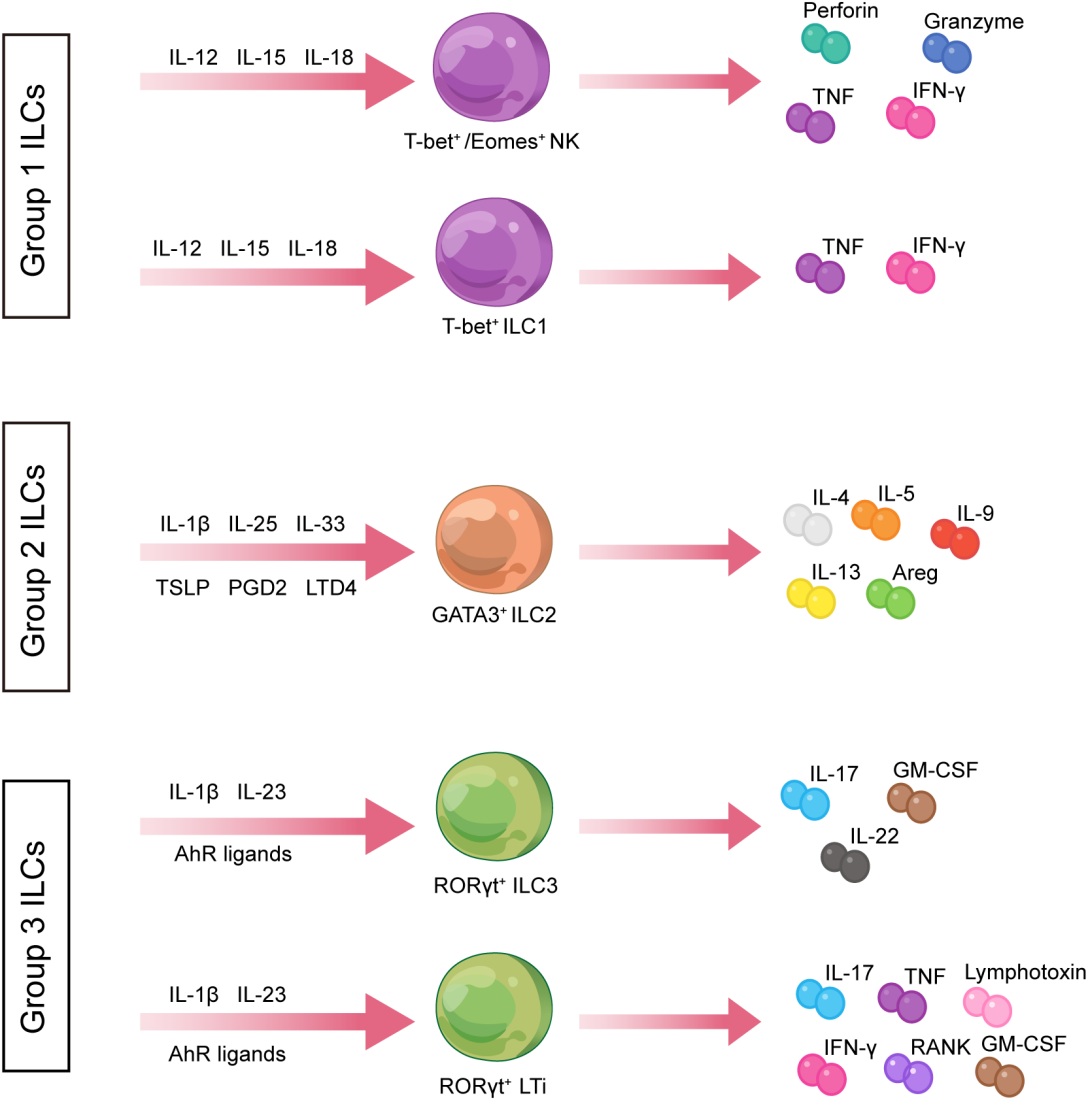
Classification of innate lymphoid cells. ILCs are divided into three groups. ILC1s produce type 1 cytokines such as TNF and IFN-γ and express T-bet in response to IL-12, IL-15, and IL-18 stimulation. ILC2s are defined by the expression of RORγt and secretion of Th2-associated cytokines such as IL-4, IL-5, and IL-13. ILC3s express GATA3 and produce IL-17A and IL-22 in the stimulation setting by IL-1β, IL-23, and AHR ligands. AHR, aryl hydrocarbon receptor; Areg, amphiregulin; GM-CSF, granulocyte–macrophage colony-stimulating factor; IFN-γ, interferon-gamma; IL, interleukin; ILCs, innate lymphoid cells; LTD4, leukotriene D4; NK, natural killer; PGD2, prostaglandin D2; RANK, receptor activator of nuclear factor kappa B; Th, T helper; TNF, tumor necrosis factor; TSLP, thymic stromal lymphopoietin.

The ILC family comprises a group of immune cells with pleiotropic functions, which lack somatic rearrangements of immune receptor genes characteristic of T and B cells (12). In the early phases of the study, it was customary to group ILCs into three major categories, with different functions for each subset, namely, natural killer (NK) cells, RORγt^+^ ILCs, and type 2 ILCs (13). Subsequently, the International Union of Immunological Societies (IUIS) approved the classification of ILCs into five subpopulations, namely, NK cells, ILC1s (group 1 ILCs), ILC2s (group 2 ILCs), ILC3s (group 3 ILCs), and lymphoid tissue-inducing (LTi) cells, based on the various developmental trajectories and transcription factors expressed by ILCs (12, 14, 15) (**Figure 1**).

NK cells are cytotoxic lymphocytes with a shorter half-life than B and T lymphocytes and occur more frequently in the circulatory system (16). NK cells can directly induce the death of tumor and virus-infected cells without specific immunization, thereby controlling intracellular pathogens (17, 18). NK cells depend on the IL-15 developmental pathway, with differential expression of GATA binding protein 3 (GATA3) and IL-7 receptor α-chain (CD127) (19, 20). Based on the relative expression of surface markers CD16 and CD56, NK cells in human peripheral blood could be subdivided into CD56^bright^ CD16 ^−^ and CD56^dim^ CD16^+^ NK cells (21). NK cell subpopulations differ in their cytolytic activity and cytokine production capacity. Vosshenrich et al. speculated that the two CD56 NK cell subsets in humans might share characteristics with various NK cells generated by the bone marrow and thymic NK cell pathways in mice (20).

The ILC1s monitor the immune system and defend the host, and they are often non-cytotoxic or weakly cytotoxic (12). ILC1s and NK cells differ in the production and dependence of transcription factors (11, 22). Zhang et al. proposed that NK cells are defined by high levels of co-expression of T-bet and eomesdermin (Eomes), whereas ILC1s are defined by the single expression of T-bet or Eomes (23). Similar to NK cells, ILC1s are developmentally reliant on T-box transcription factor (T-bet) and release type I cytokines such as interferon-gamma (IFN-γ) and tumor necrosis factor (TNF) (12). T-bet has been shown to bind to the promoters of protein-coding genes in Th1 cells, activating many critical genes in the Th1 cell response, suggesting that ILC1s may contribute to the Th1 cell response (24). Unlike NK, ILC1s are the first and primary producers of IFN-γ *in vivo* during the early stages of viral infection and do not require IL-18 signaling to optimize IFN-γ production (25). ILC1s produce optimal IFN-γ in a signal transducer and activator of transcription 4 (STAT4)-dependent manner via tissue-resident X-C motif chemokine receptor 1-positive conventional dendritic cells (XCR1^+^ cDC1), thereby limiting viral replication at the initial site of infection (25). Furthermore, RNA-sequencing analysis suggests that *Itgb3* (encoding CD61) and *Cd200r1* (encoding CD200r1) may be new, reliable specific markers to distinguish peripheral tissue-resident ILC1s from circulating NK cells, providing new insights for future studies (25).

ILC2s are usually considered substantial members of the ILC family involved in innate immune responses and regulation of tissue homeostasis (26). RORα and GATA3 (27), which are ILC2-specific transcription factors, are required for ILC2 formation. ILC2s express IL-7Rα, CD45 (28), BCL11B, and GFI1 (29), and their distinctive characteristic is the secretion of Th2-associated cytokines such as IL-4, IL-5, IL-9, IL-13, and amphiregulin (AREG) (10).

ILC3s depend on RORγt for their functional development, expressing natural cytotoxicity receptors (NCRs) and the surface marker IL-23R (12). According to the expression of NCR NKp44, ILC3s could be categorized into two main subgroups: NCR ^−^ ILC3s and NCR^+^ ILC3s. NCR ^−^ ILC3 equivalent Th17 cells express RORγt and produce IL-17A/IL-22, and NCR^+^ ILC3 equivalent Th22 cells express transcripts of RORγt and aryl hydrocarbon receptor (AHR) and produce only IL-22 (30). ILC3s modulate adaptive Th17 cell responses and produce Th17-related cytokines such as IL-17 and IL-22 (31).

LTi cells are crucial for secondary lymphoid organ formation during embryogenesis and act in T- and B- cells’ development, activation, and function (32). It is essential for LTi cells to express the chemokine receptors CXCR5 and CXCR6 in order to differentiate to the next stage (33). Additionally, LTi cells are similar to ILC3s, expressing RORγt markers and releasing cytokines that overlap with ILC3, such as IL-17 and IL-22 (34). As a crucial transcription factor for developing ILC3 progenitors, the promyelocytic leukemia zinc finger (PLZF) has a defining role in innate lymphocyte lineage differentiation (35). PLZF expression is not required to form LTi cells, although ILC3s are (33, 36).

## 3 ILCs in skin tissue

ILCs are widely distributed in various organs and tissue types in the human body (37, 38). ILCs are usually preferentially enriched in barrier tissues, such as the skin, intestine, and lung, which facilitate the maintenance of barrier function and response to tissue-derived signals (37). Our understanding of the functional features of skin ILCs is still developing compared with those of the lung and gut (11); thus, certain traits of skin ILCs will be discussed.

**Figure 2:**
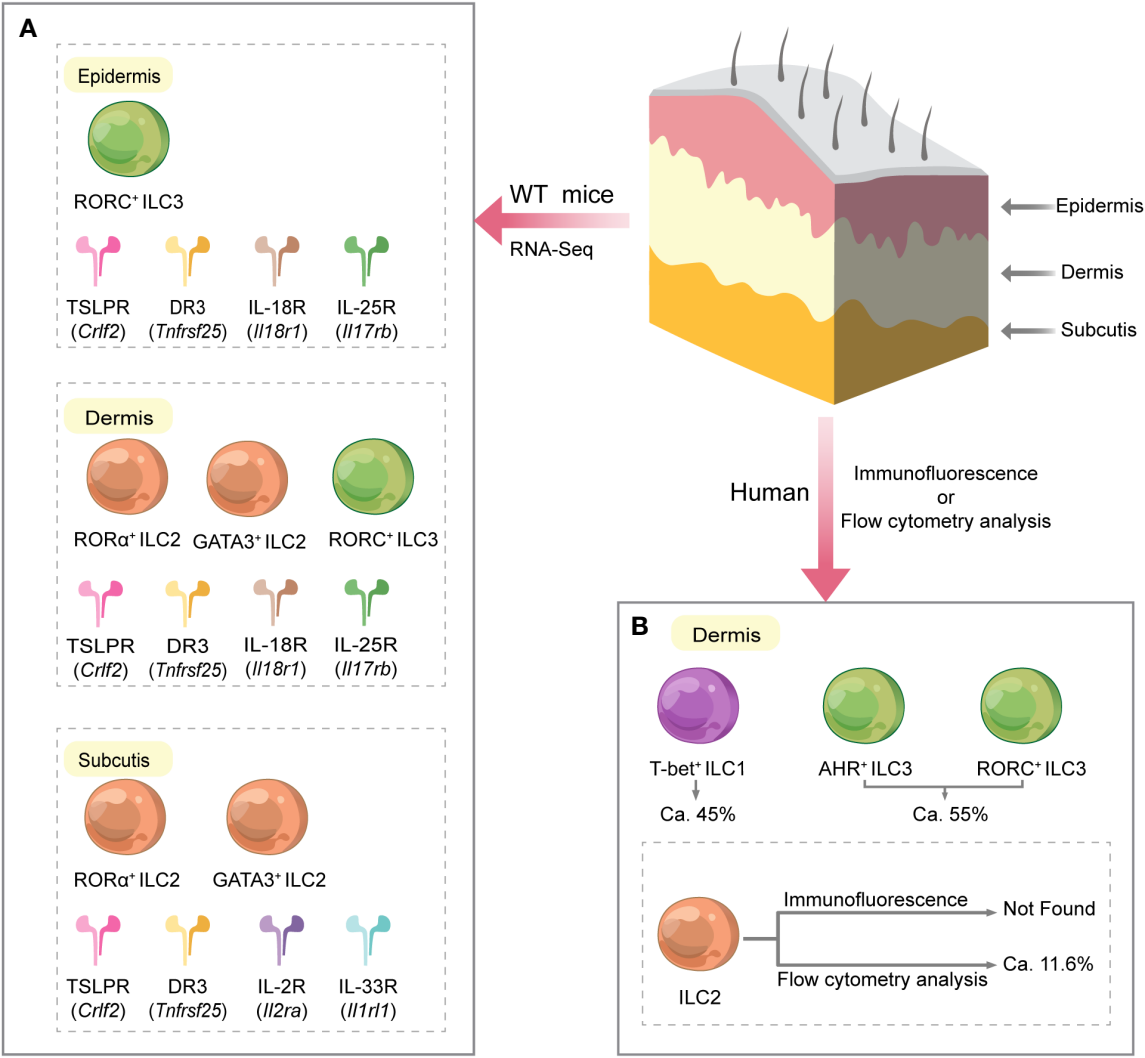
Different distribution of ILCs and their related receptors in various layers of normal mouse and human skin. **(A)** In mice, the subcutaneous and epidermal layers were highly enriched in genes characteristic of ILC2s and ILC3s, respectively, while ILC2s and ILC3s characterized the dermis. Furthermore, mouse skin RNA sequencing studies showed that ILCs in all skin layers expressed *Crlf2* (encoding the TSLPR subunit) and *Tnfrsf25* (encoding DR3). Dermal ILCs and epidermal ILCs highly express *Il18r1* (encoding IL-18R subunit) and *Il17rb* (encoding IL-25R). *Il1rl1* (encoding IL-33R subunit) and *Il2ra* (encoding IL-2R subunit) were significantly expressed on subcutaneous ILCs. **(B)** In humans, ILCs are only present in the dermis of normal skin. ILCs in the dermis are mainly composed of ILC1s and ILC3s. Flow cytometry data showed that among all dermal ILCs, ILC1s accounted for approximately 45%, ILC3s for approximately 55%, and ILC2s for approximately 11.6%. However, GATA3^+^ ILC2 was not detected in skin tissue sections by immunofluorescence. DR3, death receptor 3; IL, interleukin; IL-2R, IL-2 receptor; IL-18R, IL-18 receptor; IL-25R, IL-25 receptor; IL-33R, IL-33 receptor; ILC, innate lymphoid cell; T-bet, T-box transcription factor; TSLPR, thymic stromal lymphopoietin receptor; WT, wild type.

The skin is a mechanical and biological barrier for the body, protecting epithelial integrity and maintaining homeostasis. Anatomically, the skin consists of avascular epidermis, dermis, and subcutaneous tissue (39), each layer with specific morphological and physiological functions. Furthermore, the presence of ILCs in the skin is related to the host species and the skin layer’s location (**Figure 2**).

ILC subsets are differentially presented in various layers of mouse and human skin. NKp46 is a receptor found on the surface of NK cells from the early to late stages of differentiation. Luci et al. employed tissue immunofluorescence assay to detect NKp46 expression and discovered that the distribution of NK cells in mouse and human skin was identical at a steady state (40). This work demonstrated that NKp46^+^ CD3 ^−^ NK cells were predominantly present in the dermis and virtually absent from the epithelium, indicating that the proliferating dermal NK cells may be the source of NK cells recruited to inflamed skin during the allergic phase. In contrast, Kobayashi et al. did not find genes associated with NK cells and ILC1s by sorting and performing single-cell RNA sequencing of Lin ^−^ Thy1.2^+^ ILCs from each skin layer of wild-type (WT) C57BL/6 mice (14). Kobayashi et al. and Luci et al. used mice with the same genetic background. Still, they were controversial about the frequency of NK cells in the skin, probably related to the different technical aspects of the assay. Kobayashi et al. also revealed that in mice, the subcutaneous and epidermal layers were highly enriched in genes characteristic of ILC2s and ILC3s, respectively, and the dermis was characterized by both ILC2s and ILC3s (14).

Alkon et al. reported that ILCs from AD skin frequently co-expresses type 2 (*GATA3* and *IL13*) and type 3/17 (*RORC*, *IL22*, and *IL26*) molecular signatures at the single-cell level and can rapidly change their molecular, immunophenotypic, and functional characteristics upon cytokine stimulation, participating in host defense or promotion of disease onset (41). Reynolds et al. showed by single-cell RNA sequencing that ILCs in the epidermis and dermis of AD patients and normal healthy subjects could be classified into four subgroups, namely, ILC1/3, ILC2, ILC1/NK, and NK, with ILC2s (*IL7R*, *PTGDR2*, and *GATA3*) having the most distinct signature (42). Additionally, Brüggen et al. demonstrated that very sparse ILCs are present in the upper dermis of normal human skin with an algorithm-based *in-situ* analysis technique, while the hypodermal areas and epidermis are almost devoid of ILCs (43). Using immunofluorescence, they found that the ILC population in the upper dermis was dominated by ILC1s, followed by AHR^+^ ILC3s, and no GATA3^+^ ILC2s were observed (43). Instead, flow cytometry analysis revealed the presence of ILC2s in normal human skin cell suspensions, accounting for approximately 10% of all ILCs (43). The controversial results of the two methods in this study may be related to factors such as sample collection site, cell migration, changes in cell phenotype during isolation and purification, and the sensitivity of the assay.

Additionally, single-cell RNA-sequencing studies of wild-type C57BL/6 mouse skin showed that ILCs in all skin layers expressed *Crlf2* [encoding the thymic stromal lymphopoietin (TSLP) receptor subunit] and *Tnfrsf25* [encoding death receptor 3 (DR3)] (14). Dermal and epidermal ILCs highly express *Il18r1* (encoding IL-18 receptor subunit) and *Il17rb* (encoding IL-25 receptor) (14). *Il1rl1* (encoding IL-33 receptor subunit) and *Il2ra* (encoding IL-2 receptor subunit) were significantly expressed on subcutaneous ILCs (14) (**Figure 2**). These findings suggest a layer-specific receptor expression pattern in the skin, implying that cytokine species may be different in skin layers (14), which contributes to a better understanding of the mechanisms of localization of cytokine signaling pathways in the skin.

## 4 ILCs in atopic dermatitis

### 4.1 NK cells in atopic dermatitis

As one of the innate lymphocytes, NK cells are important sentinels of the organism to operate the immune system. NK cells exert immunomodulatory functions early in the inflammatory response, mainly by forming crosstalk effects with other immune cells and secreting a large variety of cytokines, such as TNF-α, IFN-γ, GM-CSF, IL-5, IL-6, and IL-10 (44–46). Significantly, NK cells induce Th1 cells to initiate a protective reaction by releasing IFN-γ, which facilitates the maintenance or enhancement of the body’s antiviral immunity (44, 47). NK cells have been detected in the damaged skin of patients with atopic dermatitis and MC903-induced AD-like mouse models (a systemic AD-like inflammatory phenotype closely resembling human AD was induced by the topical application of MC903 to the skin) (48). It has been reported that peripheral NK cells were significantly reduced in AD patients, possibly related to chemokine-dependent NK cell recruitment from the periphery to the lesioned skin (45). C motif chemokine receptor 2 (CCR2), C-C motif chemokine receptor 5 (CCR5), and C-X-C motif chemokine receptor 3 (CXCR3) are the primary chemotactic receptors that regulate circulating NK cell migration (49).

**Figure 3:**
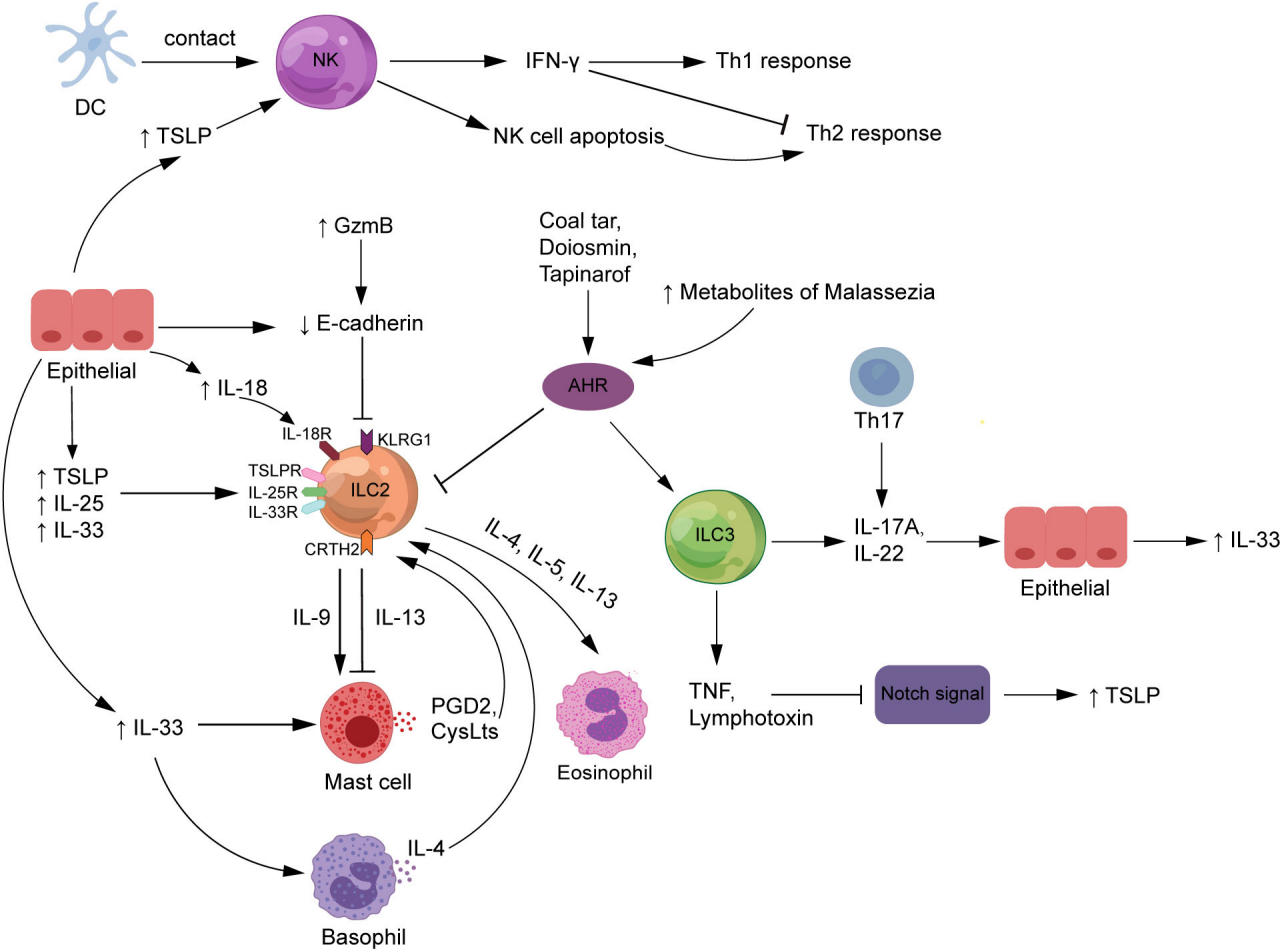
The roles of ILCs in atopic dermatitis. NK cells are stimulated by DC exposure or high TSLP levels to enhance Th1 responses, attenuate Th2 responses by producing IFN-γ, and improve Th2-type immunity by promoting auto-apoptosis. The expression of TSLP, IL-33, and IL-25, released by epithelial cells and serve as major ILC2 activators, is increased in AD patients. The interaction of ILC2s with other innate immune cells, such as mast cells and basophils, is critical to the complex mechanics of AD. Also, ILC3s release IL-17A or IL-22, which contribute to the pathogenesis of AD. Increased GzmB expression and FLG deficiency in AD patients both enhance E-cadherin cleavage, which inhibits the interaction between E-cadherin and the KLRG1 receptor expressed on ILC2s, which would strengthen the body’s ILC2-related response. ILCs may trigger TSLP secretion by producing TNF and lymphotoxin to downregulate Notch signaling. In addition, diosmin, coal tar, and tapinarof inhibit the action of ILC2s but promote the function of ILC3s by activating the AHR signaling pathway. AHR, aryl hydrocarbon receptor; CRTH2, chemoattractant receptor-homologous molecule expressed on Th2 cells; CysLts, cysteinyl leukotrienes; DCs, dendritic cells; FLG, filaggrin; GzmB, granzyme B; IFN-γ, interferon-gamma; IL, interleukin; ILCs, innate lymphoid cells; KLRG1, killer cell lectin-like receptor G1; NK, natural killer; PDG2, prostaglandin D2; Th, T helper; TNF, tumor necrosis factor; TSLP, thymic stromal lymphopoietin.

Bi et al. demonstrated that NK cell activation inhibited ILC2 amplification and cytokine production *in vitro* and *in vivo* and that this modulation was predominantly mediated by IFN-γ (50). Mature CD56^dim^ NK cells were recovered in most AD patients after dupilumab treatment, indicating that the NK cell deficiency in AD patients was reversible by the blockade of type 2 cytokines (48). Moreover, in a mouse model of NK cell-deficient AD (AD-like disease induced in *Il15* ^−/−^ mice by application of MC903), the reduction in NK cell numbers was restored by dupilumab as a Th2 cytokine blocker, suggesting that NK cells may contribute to suppressing the type 2 inflammation in AD (48). The inflammatory effects of ILC2s may be inhibited by IFN-γ released by NK cells, although more validation in animal models and patients at various illness stages is required. Mack et al. demonstrated that NK cell deficiency in mice could lead to the deterioration of pathogenic ILC2 responses *in vivo*, assuming that the NK cell–ILC2 inhibition axis may be a potential regulatory mechanism in the skin barrier (48) (**Figure 3**). This finding suggests that defects in NK cell numbers or function lead to type 2 inflammation and skin damage, further suggesting that NK cells may be closely associated with AD development.

Furthermore, NK cells also contribute to the body’s protective immunity. As important antigen-presenting cells in the immune response, NK cells can selectively edit dendritic cells (DCs) by killing immature DCs while retaining mature ones, which is required for adaptive immune responses to be initiated successfully (51). NK cells undergo close contact with DCs in the affected tissues of AD patients, suggesting that NK cells are induced to become preferential targets for apoptosis after exposure to activated monocytes, which also enhances the deviation of immune response from Th1 toward Th2 type and contributes to microbial infection (45, 52, 53). However, the activation signals that trigger natural killer cell death *in vivo* are currently unknown. TSLP is an epithelial cell-derived cytokine that is one of the key factors driving the development of the vicious cycle of inflammation in AD (54). TSLP could activate DCs to promote Th2 immune responses and has been reported to act directly on NK cells expressing TSLPR and IL-7Rα to produce IL-13, suggesting that TSLP may be a key factor in the role of NK cells in AD development (55) (**Figure 3**).

Maintaining a relatively stable number and function of NK cells *in vivo* is critical to the progression of AD, and a clearer understanding of the specific pathways of NK cells in the pathogenesis of AD may provide new strategies for AD.

### 4.2 ILC2s: the critical ILCs in atopic dermatitis

Kim et al. first found the presence of skin-derived ILC2s in healthy human skin (56). They observed a significant increase in the frequency of ILC2s in lesional AD skin compared with healthy control skin by flow cytometry, suggesting that ILC2s perform a crucial function in developing skin inflammation. ILC2s, generally considered to be the most important ILC subtype in AD pathogenesis, promote the development of Th2 cells by producing characteristic cytokines, such as IL-13 and IL-5, and it has been demonstrated that ILC2 deficiency leads to severe defects in Th2 cell immune responses (57). Interestingly, Alkon et al. showed that cutaneous ILC2 in patients with AD can have cytokine transcripts characteristic of type 17 and/or type 3 immunity that can co-produce cytokines such as IL-5, IL-13, IL-22, and IL-17A (41).

#### 4.2.1 Modulators of activated ILC2s in atopic dermatitis

It is well known that TSLP, IL-33, and IL-25 are major activators of ILC2s, and all of these cytokines have been reported to be elevated in the skin of AD patients (29).

ILC2s express receptors for TSLP, IL-33, and IL-25, all of which have a cascade of regulatory and recruitment effects on ILC2s in AD (58). TSLP, IL-33, and IL-25 can activate ILC2s to secrete various pro-inflammatory factors to induce the development of AD, and this effect can be amplified by the stimulation of allergens such as house dust mite (HDM) extraction (29, 58) (**Figure 3**). Studies have shown that TSLP can interact directly with T cells from AD patients to enhance Th2 responses by promoting the proliferation of IL-4-producing cells and secretion of IL-4 (59). IL-33 facilitates the survival and function of mast cells and basophils, which may be related to disrupting the skin barrier in AD patients and accumulating these innate immune cells in the skin lesions (60).

Of note, the relative magnitude of the contribution of IL-33 and TSLP in the inflammatory response in AD remains controversial. Kim et al. showed that AD development in mice is heavily dependent on TSLP but independent of IL-33 and IL-25 (56). In the AD-like model of inflammation, deficiency of the TSLP receptor gene in mice significantly reduced the frequency and absolute number of ILC2s, while the IL-25 or IL-33 receptor gene deficiency did not affect the ILC2 response (56). In contrast, Salimi et al. suggested that adding IL-25 and IL-33 but not TSLP increases ILC2s (58). In parallel experiments where the TSLP, IL-33, and IL-25 receptor genes were each individually knocked out in MC903-induced AD mice, the number of ILC2 was sequentially reduced in skin lesions of these mice compared with wild-type mice (58). One explanation for this contradictory finding could be related to the differences in the genetic background of the mice in the two laboratories.

Indeed, most skin ILC2s have low receptor expression for the epithelial cytokines IL-33, IL-25, and TSLP and are primarily activated by IL-18, which is highly expressed in skin ILC2s (61). Ricardo-Gonzalez et al. showed that skin ILC2s can respond to IL-18 to produce type 2 inflammatory cytokines. In AD-like skin inflammation, IL-18-deficient mice had reduced amounts of IL-5- and IL-13-producing ILC2 in skin tissues compared with WT mice (61). Serum IL-18 was elevated in AD patients compared with healthy controls and correlated with disease severity (62), implying that targeting IL-18 may improve type 2 immune activation in AD.

#### 4.2.2 Interaction of ILC2s with other innate immune cells in atopic dermatitis

The interaction of ILC2s with other innate immune cells, such as mast cells and basophils, is key to the etiology of complex AD (**Figure 3**). Mast cells produce and release various pro-inflammatory mediators such as histamine, chemokines, and cytokines, pivotal in the IgE-mediated skin wheal reaction and its associated AD pruritus (63). Studies have shown that the proportion of mast cells containing TNF-α, IL-4, IL-6, and CD30 ligand immunoreactive is higher in AD lesions than in non-lesioned skin (63). Intravital multiphoton microscopy revealed that normal murine dermal ILC2s (dILC2s) preferentially reacted with skin-resident mast cells and had pro- and anti-inflammatory properties (64). The almost exclusive production of IL-13 by dILC2 in the skin may be associated with AD. The results of *in-vitro* experiments showed that co-incubation of mast cells with recombinant IL-13 had a dose-dependent inhibitory effect on the release of IgE-dependent cytokines from mast cells, suggesting that dILC2 has the potential to modulate mast cell function through IL-13 production (64). However, once stimulated by inflammation, dILC2 exerted a pro-inflammatory effect and was able to promote eosinophil infiltration and mast cell activation in the skin (64). Additionally, a substantial amount of human and mouse research data supports the idea that IL-9 acts as a Th2 cytokine to stimulate type 2 immune responses (65). IL-9 mRNA expression was significantly increased in AD patients’ peripheral blood and skin lesions compared with normal subjects (66), and polymorphisms in IL-9 and IL-9 receptor genes were associated with the AD phenotype (67). IL-9 enhances mast cell proliferation and function and is produced mainly by T cells but also by ILC2s, mast cells, and eosinophils (68). These findings suggest that ILC2s and mast cells may crosstalk through IL-9 in AD pathogenesis.

Flow cytometry data and fluorescence microscopy images indicated that basophils and ILC2s were enriched and aggregated near inflamed lesions of AD patients and AD mouse models (69, 70). Interestingly, Mashiko et al. found that the frequency of basophils in skin lesions of AD patients was positively correlated with cutaneous ILC2s and negatively correlated with circulating ILC2s, suggesting that basophils may promote the migration of circulating ILC2s to the skin of AD patients (71). Moreover, the temporal analysis showed that on day 4 of MC903 treatment, the frequency and the absolute number of basophils in mouse skin lesions were significantly higher compared with controls but not ILC2s, suggesting that the basophil response preceded the ILC2 response in the context of AD-like inflammation (69). Studies have shown that IL-4 from basophils is required for the proliferation of ILC2s and the development of related responses in skin inflammation (69, 72). To determine the role of basophils, an anti-FcϵRI monoclonal antibody (MAR-1) was used to deplete basophils (73). Imai et al. systematically conditioned the clearance of basophils using MAR-1 or Bas-TRECK mice [basophils in mice are specifically depleted by a toxin receptor-mediated conditional cell knockout (TRECK) system] and found that ILC2 responses were suppressed along with relief of AD-like inflammation, suggesting that ILC2s mediate the innate immune response in conjunction with basophils in AD (72). The exact mechanism by which cross-regulation between ILC2s and basophils occurs in AD remains unclear, and other upstream innate cellular mechanisms are largely unexplored.

### 4.3 Role of ILC3s in atopic dermatitis

Type 2 cytokines are usually considered to have a substantial role in AD development, whereas evidence indicates that ILC3s operate in a pathogenic function in AD through the secretion of IL-17A and IL-22 (43, 74) (**Figure 3**). The percentage of IL-17^+^ T cells in peripheral blood was significantly higher in AD patients compared with healthy controls and correlated with the severity of the disease (75). Furthermore, immunohistochemical results revealed a significant infiltration of IL-17^+^ T cells in the dermis of acute AD lesions, indicating that IL-17 is the mediator of AD inflammation (75). Nakajima et al. found that IL-17A deficiency in mice alleviated the development of AD-like lesions and attenuated the expression levels of Th2 chemokines (76). IL-17A induces Th2-type immune responses in the AD mouse model, but drawing human conclusions from this model may be challenging.

Traditionally, Th17 cells are considered the primary source of IL-17, but recent studies have shown that IL-17 produced by ILC3s has a potentially important function in skin inflammation (11). Using *in-situ* mapping, Bruggen et al. discovered that skin lesions from AD patients had a significantly higher number of AHR^+^ ILC3s than those of healthy human skin (43). Similarly, Kim et al. employed flow cytometry to uncover higher levels of ILC3s in the peripheral blood of AD patients compared with healthy controls and increased in HDM-treated C57BL/6 mice AD model (an allergen-induced mouse model with phenotypes similar to human AD) (77). These findings suggest the contribution of ILC3s to the development of AD. Kim et al. sorted ILC3s from skin-draining lymph nodes and spleens in HDM-induced AD mice and injected them subcutaneously into recipient mice (C57BL/6 mice). The results showed that the adoptive transfer of ILC3s in mice accelerated the development of AD inflammation, as evidenced by increased epidermal thickness and inflammatory granulocyte infiltration, implying that ILC3s alone are sufficient to exacerbate the symptoms of AD (77). Likewise, data from co-culture cell experiments indicate that IL-17A secreted by ILC3s triggers the synthesis of IL-33 by skin cells, promoting a type 2 response (77).

Healthy people’s blood and skin contain NCR ^−^ ILC3s, which can develop into NCR^+^ ILC3s and release IL-22 (78–80). Unlike psoriasis, IL-22 expression is more dominant than IL-17 in AD lesions (74). Clinical and animal studies have shown that IL-22 expression is significantly upregulated in AD-like skin lesions, with an important link between the skin barrier and adaptive immunity (81, 82). In a randomized, double-blind, placebo-controlled trial, fezakinumab (an anti-IL-22 monoclonal antibody) had good efficacy and safety in treating adult patients with moderate-to-severe AD, confirming IL-22 as a crucial driver of AD (83). In addition, ustekinumab, a monoclonal antibody that binds to the p40 subunit of IL-12 and IL-23 and limits the progression of the Th17 inflammatory immune response, is controversial in the clinical efficacy of AD patients (84). A patient with long-standing AD showed remarkable improvement following ustekinumab treatment (84). Contrarily, one case report indicated that AD was aggravated in a patient with psoriasis who had a history of childhood atopy while receiving ustekinumab medication, raising the possibility that ustekinumab treatment may be linked to AD relapse (85). These clinical trials indicate that biologics targeting ILC3-associated cytokines may be a new approach to treating AD, but caution and more trial data are still needed.

## 5 Possible ILC-related signaling pathways in atopic dermatitis

The Notch signaling pathway has been reported to be an important player in the biology of ILCs. Moreover, ILC2s and ILC3s are significantly elevated in skin lesions of AD patients compared with normal human skin. ILC2 cells express KLRG1 and CRTH2, and ILC3 cells express AHR. Therefore, four possible signaling pathways related to ILCs in AD are discussed below.

### 5.1 The Notch signaling pathway

Skin ILCs are bona fide tissue-resident immune cells that control barrier homeostasis and maintain a healthy microbial ecology (14). During homeostasis, epidermal and dermal ILCs inhibit sebocyte proliferation and enhance commensalism of Gram-positive cocci by expressing TNF and lymphotoxin downregulating Notch signaling (14). ILCs may be upstream signals of the Notch signaling pathway that regulate mucosal barrier immunity and skin surface microbial homeostasis in AD (**Figure 3**).

Notch signaling is one of the typical pathways of epithelial differentiation and regulates the proliferation, differentiation, migration, and apoptosis of epidermal cells together with other cellular pathways *in vivo* (86). Notch signaling plays a pivotal role in ensuring normal skin development and differentiation and maintaining skin barrier function, and its abnormal disruption will induce the development of inflammatory skin diseases (86, 87). Adult mice lacking Notch signaling produce large amounts of TSLP, which caused an AD-like inflammatory response, suggesting that enhanced Notch signaling may suppress TSLP production in AD (88). Notch receptors were strongly expressed in skin tissues of psoriasis and lichen planus patients; however, they were significantly downregulated in skin lesions of AD patients as compared with healthy controls, implying that the regulation and signaling of Notch receptors are more closely related to AD than to psoriasis and lichen planus (88).

### 5.2 The AHR signaling pathway

Substantial amounts of AHR^+^ ILC3s have been reported in skin lesions of AD patients, suggesting that AHR expression may play an important role in the pathogenesis of AD (43). AHR is a ligand-dependent transcription factor that senses environmental changes. AHR could be activated by a wide range of endogenous and exogenous molecules, regulate gene expression *in vivo*, maintain tissue barriers in barrier organs, and control commensal microbiota (89–91). Growing evidence suggests that AHR can control ILCs *in vivo* (**Figure 3**).

The maintenance, survival, and function of ILC3s depend on AHR expression, which is also crucial for the defense and homeostasis of the host intestinal tissues (92, 93). Studies have shown that AHR deficiency reduces the number of intestinal RORγt^+^ ILCs, and AHR is necessary for their survival and the generation of IL-22 under homeostatic conditions (94). In addition, the amount of AHR protein and mRNA expressed in ILC2s in the mouse intestine is higher than both ILC progenitors and other mature ILCs (95). In contrast to promoting the maintenance of ILC3s, AHR inhibits the function of ILC2s, suggesting that the host regulates intestinal ILC2–ILC3 homeostasis by engaging in the AHR pathway (95). Craig et al. reported that multiple factors in the pathogenesis of AD involve dysbiosis of the gut flora and increased intestinal permeability (96), suggesting a communication mechanism between the skin and the gut in AD patients. There may be pathways in the gut of AD patients where AHR signaling regulates ILC homeostasis, and the details of the molecular mechanisms remain poorly understood.

AHR is also highly expressed on skin cells, especially in the stratum corneum, and can maintain skin homeostasis by regulating epidermal barrier protein genes (97). Diosmin is considered a potential AHR agonist from a natural product that restores the skin barrier of human keratin-forming cells by upregulating the AHR pathway to enhance the expression of skin barrier proteins such as filoproteins and loricrin and their upstream regulators (98). In addition, coal tar, an ancient topical treatment for dermatological disorders, induces keratin-forming cell-derived antimicrobial peptides by activating the AHR signaling pathway, which is beneficial in restoring the damaged skin barrier in AD patients (99). Tapinarof, a natural activator of AHR, has been considered safe and effective in clinical trials to improve symptoms in AD patients (100, 101). *Malassezia* generates cultured metabolites as AHR ligands and may activate the AHR pathway, causing aberrant keratinization and scaling frequently observed in dermatological conditions (97). *Malassezia* is known to be one of the most common fungi associated with AD (102), indicating that there may be a mechanism for *Malassezia* activation of AHR signaling in AD involved in skin barrier defects in patients.

Diosgenin, coal tar, and tapinarof have all been shown to alleviate skin lesions in AD patients, while *Malassezia* metabolites have been shown to worsen the skin barrier by stimulating the AHR pathway. Clarifying the cell-intrinsic function of AHR in ILCs is crucial to develop a potential therapeutic strategy for AD, given that AHR and ILCs are closely linked and affect how AD develops.

### 5.3 The ILC2–KLRG1–E-cadherin axis

The killer cell lectin-like receptor G1 (KLRG1) is an inhibitory receptor belonging to the C-type lectin family, mainly expressed in NK cells and T cells, and its main ligands are E-cadherin and N-cadherin (103). KLRG1 engagement inhibits protein kinase B (AKT) phosphorylation, leading to proliferative dysfunction of T cells and NK cells (104). Alkon et al. showed that most ILCs in the skin lesions of AD patients belonged to the CRTH2^+^ ILC2 subgroup (41). ILC2s were enriched in the skin of AD acute lesions, and KLRG1 expression on these cells was markedly increased compared with ILC2s in healthy and unaffected skin (58). Also, KLRG1 expression was further upregulated by IL-33 or TSLP as activators of ILC2s (58), supporting the connection between the function of ILC2s and KLRG1 expression. It was shown that activated skin-resident ILC2s express high levels of KLRG1, which significantly inhibit the function of ILC2s upon interaction with E-cadherin, as evidenced by the downregulation of the expression of GATA3, as well as reduced production of IL-13, IL-5, and AREG (58). This indicated that downregulation of E-cadherin may interrupt this inhibitory signal, prompting ILC2s to release more type 2 cytokines through this new barrier-sensing mechanism and even unrestricted ILC2 proliferation and cytokine expression (**Figure 3**).

E-cadherin, as one of the important ligands of KLRG1, is a central adhesion molecule widely found in normal epithelial cells, keratinocytes, and Langerhans cells and is pivotal for maintaining epithelial cell integrity (105). E-cadherin has been reported to be reduced in damaged skin of individuals with AD disease (58, 106), indicating that the absence of this epidermal linker protein may enhance the generation of more type 2 cytokines by ILC2s in AD. After shRNA knockdown of the FLG gene, human keratin-forming cells produce less E-cadherin, demonstrating that FLG gene abnormalities may be the reason for the decreased expression of E-cadherin in lesional skin of AD patients (58).

Furthermore, granzyme B (GzmB) abnormalities are an important factor in the decreased expression of E-cadherin in patients with AD. GzmB is a serine protease that cleaves E-cadherin, a key mediator of skin injury, inflammation, and repair (107, 108). Plasma GzmB concentrations were significantly higher in AD patients than in healthy controls and positively correlated with pruritus and dermatitis severity (109). In contrast to non-lesional AD and healthy skin, Turner et al. showed that cell-specific GzmB immunological positivity was enhanced in the lesional AD dermis and expressed primarily by mast cells (107). *GzmB* ^−/−^ mice exhibited fewer mast cells, less severe dermatitis, and better skin barrier function compared with wild controls in an oxazolone (OXA)-induced mouse dermatitis model (OXA was repeatedly applied as a hapten to the mouse ear to cause skin inflammation similar to that of human AD), indicating that GzmB may be a potential therapeutic target for AD (107). The findings further showed that E-cadherin was reduced in the epidermis of both *GzmB ^−/−^* and WT mice with OXA dermatitis compared with control skin, and the reduction was more pronounced in WT mice compared to *GzmB ^−/−^* mice (107). In addition, immunohistochemical results showed that E-cadherin in living human skin showed lower staining intensity with tissues incubated with GzmB, and preincubation of GzmB with VTI-1002, a potent and specific small-molecule inhibitor of GzmB, followed by exposure of *in-vitro* skin lessened the effect of GzmB on the detection of E-cadherin (107). The above experimental results suggest that high expression of GzmB in AD patients may lead to impaired barrier function in AD by cleaving E-cadherin.

The ILC2–KLRG1–E-cadherin axis is a novel skin barrier sensing mechanism that contributes to a fuller understanding of the pathogenesis of impaired skin barrier function in AD. Reducing the expression of GzmB and promoting the binding of E-cadherin to KLRG1 in AD patients may provide practical ideas for limiting the inflammation caused by ILC2s.

### 5.4 The PGD2–CRTH2–ILC2 pathway

Prostaglandin D2 (PGD2) is the predominant prostaglandin produced by activated mast cells. As reviewed by Honda et al., the skin of AD patients produces several prostaglandins, including PGD2 (110). Inagaki et al. reported that urinary levels of PGD2 metabolites in children AD patients were essentially the same as in healthy control children, suggesting that PGD2 metabolites may not be a useful clinical indicator for assessing AD (111). Additionally, cyclooxygenase inhibitors were unsuccessful in alleviating the symptoms of AD, implying a weak association of prostaglandins with AD pathogenesis (110). However, prostaglandin receptors, as mediators of inflammation, have recently been found to play a crucial regulatory function in AD development. PGD2 has two central receptors, the D-prostanoid receptor (DP) and chemoattractant receptor-homologous molecule expressed on Th2 cells (CRTH2), which exert opposite regulatory functions at different stages of skin inflammation (112).

On the one hand, PGD2–DP signaling reduces early skin inflammation by promoting vascular endothelial barrier formation and inhibiting skin DC migration to draining LNs (112). On the other hand, CRTH2 was determined to be expressed on human ILCs and is more critical in allergic inflammation (28). PGD2–CRTH2 signaling exerts a pro-inflammatory effect in the late stages of the disease by effectively activating type 2 immune cells and the activation of ILC2s (110, 112) (**Figure 3**). The recruitment response of ILC2s to tissues is enhanced following PGD2–CRTH2 pathway activation, and their expression of the IL-33 receptor (ST2) and IL-25 receptor subunit (IL-17RA) is upregulated, promoting the production of type 2 cytokines as well as other inflammatory cytokines (113, 114). The relationship between mast cells and ILC2s in AD (110) suggests that the PGD2–CRTH2–ILC2 axis controls Th2 cell-associated inflammatory responses (113–115). In analogy to PGD2, cysteinyl leukotrienes (CysLTs) are another lipid inflammatory mediator secreted by IgE-mediated activated mast cells that exert biological functions by binding to the G protein-coupled cysteinyl leukotriene receptor 1 (CysLT1) and CysLT2 (116).

ILC2s have been demonstrated to express functional CysLT1 in both humans and animals, and CysLT1 levels in ILC2s isolated from AD patients were noticeably higher than those in healthy control subjects at both the protein and mRNA levels (117, 118). *In-vitro* experiments showed that CysLTs enhanced the activation of human ILC2s by PGD2 and epithelial cytokines, promoted the migration and survival of ILC2s, and induced the secretion of type 2 cytokines by ILC2s (118). This study further revealed that CysLTs, endogenously synthesized by human-activated mast cells, also induced IL-5 and IL-13 production by ILC2s, which was considerably but only partially inhibited by CysLT1 receptor antagonists such as montelukast (118).

Golub et al. summarized that the Notch signaling pathway could be regarded as one of the key future strategic targets for regulating the immune response of ILCs to inflammation (119). The Notch pathway has been identified as an important feature driving the KLRG1^+^ ILC2 subtype and a dominant pathway downstream of the AHR during NCR^+^ ILC3 generation (119). The development of novel AD therapeutic approaches may benefit from further research on the upstream signals that stimulate Notch receptor protein upregulation, the molecular mechanisms that activate the AHR signaling pathway to inhibit the response of ILC2s, strategies to effectively reduce the expression of GzmB or decrease the degradation of E-cadherin in AD patients, and the role of antagonizing the effect of CRTH2 on ILC2s.

## 6 Perspectives

ILCs are gradually recognized as modulators of tissue homeostasis and inflammation and will undoubtedly become an emerging key factor in AD belonging to Th2-type allergic diseases. The data suggest the presence of ILCs in the normal skin of mice and humans, and their expression varies with the skin layer. ILCs accumulate in the skin of AD patients and AD mouse models, and their function is related to the degree of inflammation. Currently, ILC2s are considered the critical subtype of pro-inflammatory ILCs in AD, contributing mainly through the secretion of many pro-inflammatory factors and crosstalk with other immune cells. As discussed above, the part of ILC3s in AD is poorly explored, and ILC3s have great potential in skin barrier function and tissue repair. In addition, ILCs are pivotal in regulating the balance between the skin surface and gut microbial bacteria in AD. The AHR signaling pathway, a critical point in holding the balance of ILC2s and ILC3s *in vivo*, can potentially become a new therapeutic target for AD.

In the last decade, numerous studies have revealed the critical role of ILCs in lung and intestinal inflammation, but the understanding of the biology of ILCs in the skin is only the “tip of the iceberg.” ILCs in skin inflammation are carried out to provide a way worthy of exploration. The following needs further study: 1) Due to the absence of cell-specific surface markers and the limited reagents available, it is still difficult to accurately differentiate ILCs from T cells. 2) It is unclear how the kind and concentration of cytokines in the microenvironment relate to the activity of ILCs and whether ILCs secrete mixed cytokines like Th cells in AD. 3) Although the upstream activation signals of ILCs are known to be associated with signaling pathways in inflammatory diseases, the precise mechanisms by which they interact with other immune or non-immune cells remain to be explored in depth. 4) Since ILCs contribute to wound healing and infection resistance, it remains unclear whether treatment targeting ILC depletion disrupts the mucosal homeostasis of the patient and the relationship between the microbiota and ILCs of the skin and gut.

Strategies to address the above issues may focus on the following areas. 1) To determine cell-specific surface markers and the tissue distribution of each human ILC subpopulation, emerging technologies such as mass spectrometry, flow cytometry, and single-cell analysis methods could be used to analyze the proteomic, transcriptional, and genomic changes in ILCs (120). 2) It will be easier to induce and maintain ILCs *in vitro* with a better understanding of their origin and maintenance, enabling the execution of pertinent cellular experiments to further explore the relationship between ILCs and cytokines. 3) The rational application of dynamic *in-vivo* real-time imaging tools to study the trafficking mechanisms of ILCs in various AD mouse models will improve our understanding of the immune networks and signaling pathways associated with human diseases (120). 4) To explore the adverse effects of targeting depleted ILCs to treat AD patients, detailed information on the mechanisms of ILCs in the skin and intestinal mucosa of AD patients should be studied, which requires numerous animal experiments and clinical trials to gather necessary scientific evidence.

Collectively, although much evidence suggests that alterations in the phenotype and function of ILCs are inextricably linked to the development of AD, the picture of the role of ILCs in AD remains unclear. Understanding the biological and regulatory mechanisms in the epithelial immune barrier of ILCs will pose a significant research challenge in the future. The current review will provide insight into the pathogenesis of AD and may help develop safe and effective treatment strategies for patients with refractory AD.

## Author contributions

HJ: Writing – original draft. HW: Writing – original draft. DZ: Writing – review & editing.

## References

[1] Langan SM Irvine AD Weidinger S. Atopic dermatitis. Lancet (2020) 396(10247):345–60. doi: 10.1016/s0140-6736(20)31286-1 32738956

[2] Laughter MR Maymone MBC Mashayekhi S Arents BWM Karimkhani C Langan SM. The global burden of atopic dermatitis: lessons from the global burden of disease study 1990-2017. Br J Dermatol (2021) 184(2):304–9. doi: 10.1111/bjd.19580 33006135

[3] Shirley M. Dupilumab: first global approval. Drugs (2017) 77(10):1115–21. doi: 10.1007/s40265-017-0768-3 28547386

[4] Baghoomian W Na C Simpson EL. New and emerging biologics for atopic dermatitis. Am J Clin Dermatol (2020) 21(4):457–65. doi: 10.1007/s40257-020-00515-1 32323259

[5] Narla S Silverberg JI Simpson EL. Management of inadequate response and adverse effects to dupilumab in atopic dermatitis. J Am Acad Dermatol (2022) 86(3):628–36. doi: 10.1016/j.jaad.2021.06.017 34126094

[6] Sun Z Vattepu R Zhang S. Chemokines and innate lymphoid cells in skin inflammation. Cells (2021) 10(11):3074. doi: 10.3390/cells10113074 34831296 PMC8621478

[7] Mjösberg J Eidsmo L. Update on innate lymphoid cells in atopic and non-atopic inflammation in the airways and skin. Clin Exp Allergy (2014) 44(8):1033–43. doi: 10.1111/cea.12353 24912880

[8] Walker JA Barlow JL McKenzie AN. Innate lymphoid cells–how did we miss them? Nat Rev Immunol (2013) 13(2):75–87. doi: 10.1038/nri3349 23292121

[9] Kim BS. Innate lymphoid cells in the skin. J Invest Dermatol (2015) 135(3):673–8. doi: 10.1038/jid.2014.401 PMC4556524 25339380

[10] Zhou S Li Q Wu H Lu Q. The pathogenic role of innate lymphoid cells in autoimmune-related and inflammatory skin diseases. Cell Mol Immunol (2020) 17(4):335–46. doi: 10.1038/s41423-020-0399-6 PMC7109064 32203190

[11] Kobayashi T Ricardo-Gonzalez RR Moro K. Skin-resident innate lymphoid cells - cutaneous innate guardians and regulators. Trends Immunol (2020) 41(2):100–12. doi: 10.1016/j.it.2019.12.004 PMC7364860 31948873

[12] Vivier E Artis D Colonna M Diefenbach A Di Santo JP Eberl G. Innate lymphoid cells: 10 years on. Cell (2018) 174(5):1054–66. doi: 10.1016/j.cell.2018.07.017 30142344

[13] Spits H Cupedo T. Innate lymphoid cells: emerging insights in development, lineage relationships, and function. Annu Rev Immunol (2012) 30:647–75. doi: 10.1146/annurev-immunol-020711-075053 22224763

[14] Kobayashi T Voisin B Kim DY Kennedy EA Jo JH Shih HY. Homeostatic control of sebaceous glands by innate lymphoid cells regulates commensal bacteria equilibrium. Cell (2019) 176(5):982–97.e16. doi: 10.1016/j.cell.2018.12.031 30712873 PMC6532063

[15] Krabbendam L Bernink JH Spits H. Innate lymphoid cells: from helper to killer. Curr Opin Immunol (2021) 68:28–33. doi: 10.1016/j.coi.2020.08.007 32971468

[16] Huntington ND Cursons J Rautela J. The cancer-natural killer cell immunity cycle. Nat Rev Cancer (2020) 20(8):437–54. doi: 10.1038/s41568-020-0272-z 32581320

[17] Zitti B Bryceson YT. Natural killer cells in inflammation and autoimmunity. Cytokine Growth Factor Rev (2018) 42:37–46. doi: 10.1016/j.cytogfr.2018.08.001 30122459

[18] Vivier E Raulet DH Moretta A Caligiuri MA Zitvogel L Lanier LL. Innate or adaptive immunity? The example of natural killer cells. Science (2011) 331(6013):44–9. doi: 10.1126/science.1198687 PMC3089969 21212348

[19] Vosshenrich CA Ranson T Samson SI Corcuff E Colucci F Rosmaraki EE. Roles for common cytokine receptor gamma-chain-dependent cytokines in the generation, differentiation, and maturation of nk cell precursors and peripheral nk cells in vivo. J Immunol (2005) 174(3):1213–21. doi: 10.4049/jimmunol.174.3.1213 15661875

[20] Vosshenrich CA García-Ojeda ME Samson-Villéger SI Pasqualetto V Enault L Richard-Le Goff O. A thymic pathway of mouse natural killer cell development characterized by expression of gata-3 and cd127. Nat Immunol (2006) 7(11):1217–24. doi: 10.1038/ni1395 17013389

[21] Poli A Michel T Thérésine M Andrès E Hentges F Zimmer J. Cd56bright natural killer (Nk) cells: an important nk cell subset. Immunology (2009) 126(4):458–65. doi: 10.1111/j.1365-2567.2008.03027.x PMC2673358 19278419

[22] Daussy C Faure F Mayol K Viel S Gasteiger G Charrier E. T-bet and eomes instruct the development of two distinct natural killer cell lineages in the liver and in the bone marrow. J Exp Med (2014) 211(3):563–77. doi: 10.1084/jem.20131560 PMC3949572 24516120

[23] Zhang J Marotel M Fauteux-Daniel S Mathieu AL Viel S Marçais A. T-bet and eomes govern differentiation and function of mouse and human nk cells and ilc1. Eur J Immunol (2018) 48(5):738–50. doi: 10.1002/eji.201747299 29424438

[24] Powell N Walker AW Stolarczyk E Canavan JB Gökmen MR Marks E. The transcription factor T-bet regulates intestinal inflammation mediated by interleukin-7 receptor+ Innate lymphoid cells. Immunity (2012) 37(4):674–84. doi: 10.1016/j.immuni.2012.09.008 PMC3540260 23063332

[25] Weizman OE Adams NM Schuster IS Krishna C Pritykin Y Lau C. Ilc1 confer early host protection at initial sites of viral infection. Cell (2017) 171(4):795–808.e12. doi: 10.1016/j.cell.2017.09.052 29056343 PMC5687850

[26] Imai Y. Ilc2s in skin disorders. Allergol Int Off J Japan Soc Allergol (2023) 72(2):201–6. doi: 10.1016/j.alit.2023.01.002 36842916

[27] Mjösberg J Bernink J Golebski K Karrich JJ Peters CP Blom B. The transcription factor gata3 is essential for the function of human type 2 innate lymphoid cells. Immunity (2012) 37(4):649–59. doi: 10.1016/j.immuni.2012.08.015 23063330

[28] Mjösberg JM Trifari S Crellin NK Peters CP van Drunen CM Piet B. Human il-25- and il-33-responsive type 2 innate lymphoid cells are defined by expression of crth2 and cd161. Nat Immunol (2011) 12(11):1055–62. doi: 10.1038/ni.2104 21909091

[29] Ebbo M Crinier A Vély F Vivier E. Innate lymphoid cells: major players in inflammatory diseases. Nat Rev Immunol (2017) 17(11):665–78. doi: 10.1038/nri.2017.86 28804130

[30] Rosser EC Lom H Bending D Duurland CL Bajaj-Elliott M Wedderburn LR. Innate lymphoid cells and T cells contribute to the interleukin-17a signature detected in the synovial fluid of patients with juvenile idiopathic arthritis. Arthritis Rheumatol (2019) 71(3):460–7. doi: 10.1002/art.40731 PMC7983174 30350355

[31] Hepworth MR Monticelli LA Fung TC Ziegler CG Grunberg S Sinha R. Innate lymphoid cells regulate cd4+ T-cell responses to intestinal commensal bacteria. Nature (2013) 498(7452):113–7. doi: 10.1038/nature12240 PMC3699860 23698371

[32] Zhong C Zheng M Zhu J. Lymphoid tissue inducer-a divergent member of the ilc family. Cytokine Growth Factor Rev (2018) 42:5–12. doi: 10.1016/j.cytogfr.2018.02.004 29454785 PMC6089680

[33] van de Pavert SA. Lymphoid tissue inducer (Lti) cell ontogeny and functioning in embryo and adult. BioMed J (2021) 44(2):123–32. doi: 10.1016/j.bj.2020.12.003 PMC8178546 33849806

[34] Clottu AS Humbel M Fluder N Karampetsou MP Comte D. Innate lymphoid cells in autoimmune diseases. Front Immunol (2021) 12:789788. doi: 10.3389/fimmu.2021.789788 35069567 PMC8777080

[35] Constantinides MG McDonald BD Verhoef PA Bendelac A. A committed precursor to innate lymphoid cells. Nature (2014) 508(7496):397–401. doi: 10.1038/nature13047 24509713 PMC4003507

[36] Nagasawa M Germar K Blom B Spits H. Human cd5(+) innate lymphoid cells are functionally immature and their development from cd34(+) progenitor cells is regulated by id2. Front Immunol (2017) 8:1047. doi: 10.3389/fimmu.2017.01047 28912776 PMC5583608

[37] Mao Y Tao R Cao X Bao Q Wang D Zhao Y. Innate lymphoid cells regulate radiation-induced skin damage via ccr10 signaling. Int J Radiat Biol (2020) 96(9):1157–64. doi: 10.1080/09553002.2020.1793013 32658555

[38] Ebihara T. Dichotomous regulation of acquired immunity by innate lymphoid cells. Cells (2020) 9(5):1193. doi: 10.3390/cells9051193 32403291 PMC7290502

[39] Egert M Simmering R Riedel CU. The association of the skin microbiota with health, immunity, and disease. Clin Pharmacol Ther (2017) 102(1):62–9. doi: 10.1002/cpt.698 28380682

[40] Luci C Reynders A Ivanov II Cognet C Chiche L Chasson L. Influence of the transcription factor rorgammat on the development of nkp46+ Cell populations in gut and skin. Nat Immunol (2009) 10(1):75–82. doi: 10.1038/ni.1681 19029904

[41] Alkon N Bauer WM Krausgruber T Goh I Griss J Nguyen V. Single-cell analysis reveals innate lymphoid cell lineage infidelity in atopic dermatitis. J Allergy Clin Immunol (2022) 149(2):624–39. doi: 10.1016/j.jaci.2021.07.025 PMC9130781 34363841

[42] Reynolds G Vegh P Fletcher J Poyner EFM Stephenson E Goh I. Developmental cell programs are co-opted in inflammatory skin disease. Science (2021) 371(6527):eaba6500. doi: 10.1126/science.aba6500 33479125 PMC7611557

[43] Brüggen MC Bauer WM Reininger B Clim E Captarencu C Steiner GE. *In situ* mapping of innate lymphoid cells in human skin: evidence for remarkable differences between normal and inflamed skin. J Invest Dermatol (2016) 136(12):2396–405. doi: 10.1016/j.jid.2016.07.017 27456756

[44] Moretta A Marcenaro E Parolini S Ferlazzo G Moretta L. Nk cells at the interface between innate and adaptive immunity. Cell Death Differ (2008) 15(2):226–33. doi: 10.1038/sj.cdd.4402170 17541426

[45] Luci C Gaudy-Marqueste C Rouzaire P Audonnet S Cognet C Hennino A. Peripheral natural killer cells exhibit qualitative and quantitative changes in patients with psoriasis and atopic dermatitis. Br J Dermatol (2012) 166(4):789–96. doi: 10.1111/j.1365-2133.2012.10814.x 22233261

[46] Liu M Liang S Zhang C. Nk cells in autoimmune diseases: protective or pathogenic? Front Immunol (2021) 12:624687. doi: 10.3389/fimmu.2021.624687 33777006 PMC7994264

[47] Chiossone L Dumas PY Vienne M Vivier E. Natural killer cells and other innate lymphoid cells in cancer. Nat Rev Immunol (2018) 18(11):671–88. doi: 10.1038/s41577-018-0061-z 30209347

[48] Mack MR Brestoff JR Berrien-Elliott MM Trier AM Yang TB McCullen M. Blood natural killer cell deficiency reveals an immunotherapy strategy for atopic dermatitis. Sci Transl Med (2020) 12(532):eaay1005. doi: 10.1126/scitranslmed.aay1005 32102931 PMC7433875

[49] Grégoire C Chasson L Luci C Tomasello E Geissmann F Vivier E. The trafficking of natural killer cells. Immunol Rev (2007) 220(1):169–82. doi: 10.1111/j.1600-065X.2007.00563.x PMC7165697 17979846

[50] Bi J Cui L Yu G Yang X Chen Y Wan X. Nk cells alleviate lung inflammation by negatively regulating group 2 innate lymphoid cells. J Immunol (2017) 198(8):3336–44. doi: 10.4049/jimmunol.1601830 28275135

[51] Ferlazzo G Moretta L. Dendritic cell editing by natural killer cells. Crit Rev Oncog (2014) 19(1-2):67–75. doi: 10.1615/critrevoncog.2014010827 24941374

[52] Zaniboni MC Samorano LP Orfali RL Aoki V. Skin barrier in atopic dermatitis: beyond filaggrin. Bras Dermatol (2016) 91(4):472–8. doi: 10.1590/abd1806-4841.20164412 PMC4999106 27579743

[53] Katsuta M Takigawa Y Kimishima M Inaoka M Takahashi R Shiohara T. Nk cells and gamma delta+ T cells are phenotypically and functionally defective due to preferential apoptosis in patients with atopic dermatitis. J Immunol (2006) 176(12):7736–44. doi: 10.4049/jimmunol.176.12.7736 16751421

[54] Sparber F De Gregorio C Steckholzer S Ferreira FM Dolowschiak T Ruchti F. The skin commensal yeast malassezia triggers a type 17 response that coordinates anti-fungal immunity and exacerbates skin inflammation. Cell Host Microbe (2019) 25(3):389–403.e6. doi: 10.1016/j.chom.2019.02.002 30870621

[55] Zhang Y Zhou B. Functions of thymic stromal lymphopoietin in immunity and disease. Immunol Res (2012) 52(3):211–23. doi: 10.1007/s12026-012-8264-z PMC3350568 22274860

[56] Kim BS Siracusa MC Saenz SA Noti M Monticelli LA Sonnenberg GF. Tslp elicits il-33-independent innate lymphoid cell responses to promote skin inflammation. Sci Transl Med (2013) 5(170):170ra16. doi: 10.1126/scitranslmed.3005374 PMC3637661 23363980

[57] Halim TY Steer CA Mathä L Gold MJ Martinez-Gonzalez I McNagny KM. Group 2 innate lymphoid cells are critical for the initiation of adaptive T helper 2 cell-mediated allergic lung inflammation. Immunity (2014) 40(3):425–35. doi: 10.1016/j.immuni.2014.01.011 PMC4210641 24613091

[58] Salimi M Barlow JL Saunders SP Xue L Gutowska-Owsiak D Wang X. A role for il-25 and il-33-driven type-2 innate lymphoid cells in atopic dermatitis. J Exp Med (2013) 210(13):2939–50. doi: 10.1084/jem.20130351 PMC3865470 24323357

[59] Tatsuno K Fujiyama T Yamaguchi H Waki M Tokura Y. Tslp directly interacts with skin-homing th2 cells highly expressing its receptor to enhance il-4 production in atopic dermatitis. J Invest Dermatol (2015) 135(12):3017–24. doi: 10.1038/jid.2015.318 26288354

[60] Klonowska J Gleń J Nowicki RJ Trzeciak M. New cytokines in the pathogenesis of atopic dermatitis-new therapeutic targets. Int J Mol Sci (2018) 19(10):3086. doi: 10.3390/ijms19103086 30304837 PMC6213458

[61] Ricardo-Gonzalez RR Van Dyken SJ Schneider C Lee J Nussbaum JC Liang HE. Tissue signals imprint ilc2 identity with anticipatory function. Nat Immunol (2018) 19(10):1093–9. doi: 10.1038/s41590-018-0201-4 PMC6202223 30201992

[62] Zedan K Rasheed Z Farouk Y Alzolibani AA Bin Saif G Ismail HA. Interleukin-18 and interleukin-12 in patients with atopic dermatitis: correlation with disease activity. J Clin Diagn Res JCDR (2015) 9(4):Wc01–5. doi: 10.7860/jcdr/2015/12261.5742 PMC4437144 26023628

[63] Gupta K Harvima IT. Mast cell-neural interactions contribute to pain and itch. Immunol Rev (2018) 282(1):168–87. doi: 10.1111/imr.12622 PMC5812374 29431216

[64] Roediger B Kyle R Yip KH Sumaria N Guy TV Kim BS. Cutaneous immunosurveillance and regulation of inflammation by group 2 innate lymphoid cells. Nat Immunol (2013) 14(6):564–73. doi: 10.1038/ni.2584 PMC4282745 23603794

[65] Shik D Tomar S Lee JB Chen CY Smith A Wang YH. Il-9-producing cells in the development of ige-mediated food allergy. Semin Immunopathol (2017) 39(1):69–77. doi: 10.1007/s00281-016-0605-x 27909880 PMC5225002

[66] Ma L Xue HB Guan XH Shu CM Zhang JH Yu J. Possible pathogenic role of T helper type 9 cells and interleukin (Il)-9 in atopic dermatitis. Clin Exp Immunol (2014) 175(1):25–31. doi: 10.1111/cei.12198 24032555 PMC3898551

[67] Namkung JH Lee JE Kim E Park GT Yang HS Jang HY. An association between il-9 and il-9 receptor gene polymorphisms and atopic dermatitis in a korean population. J Dermatol Sci (2011) 62(1):16–21. doi: 10.1016/j.jdermsci.2011.01.007 21371865

[68] Angkasekwinai P Dong C. Il-9-producing T cells: potential players in allergy and cancer. Nat Rev Immunol (2021) 21(1):37–48. doi: 10.1038/s41577-020-0396-0 32788707

[69] Kim BS Wang K Siracusa MC Saenz SA Brestoff JR Monticelli LA. Basophils promote innate lymphoid cell responses in inflamed skin. J Immunol (2014) 193(7):3717–25. doi: 10.4049/jimmunol.1401307 PMC4170007 25156365

[70] Ito Y Satoh T Takayama K Miyagishi C Walls AF Yokozeki H. Basophil recruitment and activation in inflammatory skin diseases. Allergy (2011) 66(8):1107–13. doi: 10.1111/j.1398-9995.2011.02570.x 21371044

[71] Mashiko S Mehta H Bissonnette R Sarfati M. Increased frequencies of basophils, type 2 innate lymphoid cells and th2 cells in skin of patients with atopic dermatitis but not psoriasis. J Dermatol Sci (2017) 88(2):167–74. doi: 10.1016/j.jdermsci.2017.07.003 28743611

[72] Imai Y Yasuda K Nagai M Kusakabe M Kubo M Nakanishi K. Il-33-induced atopic dermatitis-like inflammation in mice is mediated by group 2 innate lymphoid cells in concert with basophils. J Invest Dermatol (2019) 139(10):2185–94.e3. doi: 10.1016/j.jid.2019.04.016 31121178

[73] Muto T Fukuoka A Kabashima K Ziegler SF Nakanishi K Matsushita K. The role of basophils and proallergic cytokines, tslp and il-33, in cutaneously sensitized food allergy. Int Immunol (2014) 26(10):539–49. doi: 10.1093/intimm/dxu058 24860117

[74] Sugaya M. The role of th17-related cytokines in atopic dermatitis. Int J Mol Sci (2020) 21(4):1314. doi: 10.3390/ijms21041314 32075269 PMC7072946

[75] Koga C Kabashima K Shiraishi N Kobayashi M Tokura Y. Possible pathogenic role of th17 cells for atopic dermatitis. J Invest Dermatol (2008) 128(11):2625–30. doi: 10.1038/jid.2008.111 18432274

[76] Nakajima S Kitoh A Egawa G Natsuaki Y Nakamizo S Moniaga CS. Il-17a as an inducer for th2 immune responses in murine atopic dermatitis models. J Invest Dermatol (2014) 134(8):2122–30. doi: 10.1038/jid.2014.51 24480880

[77] Kim MH Jin SP Jang S Choi JY Chung DH Lee DH. Il-17a-producing innate lymphoid cells promote skin inflammation by inducing il-33-driven type 2 immune responses. J Invest Dermatol (2020) 140(4):827–37.e9. doi: 10.1016/j.jid.2019.08.447 31628929

[78] Teunissen MBM Munneke JM Bernink JH Spuls PI Res PCM Te Velde A. Composition of innate lymphoid cell subsets in the human skin: enrichment of ncr(+) ilc3 in lesional skin and blood of psoriasis patients. J Invest Dermatol (2014) 134(9):2351–60. doi: 10.1038/jid.2014.146 24658504

[79] Nakajima K. Critical role of the interleukin-23/T-helper 17 cell axis in the pathogenesis of psoriasis. J Dermatol (2012) 39(3):219–24. doi: 10.1111/j.1346-8138.2011.01458.x 22352845

[80] Boniface K Bernard FX Garcia M Gurney AL Lecron JC Morel F. Il-22 inhibits epidermal differentiation and induces proinflammatory gene expression and migration of human keratinocytes. J Immunol (2005) 174(6):3695–702. doi: 10.4049/jimmunol.174.6.3695 15749908

[81] Lou H Lu J Choi EB Oh MH Jeong M Barmettler S. Expression of il-22 in the skin causes th2-biased immunity, epidermal barrier dysfunction, and pruritus via stimulating epithelial th2 cytokines and the grp pathway. J Immunol (2017) 198(7):2543–55. doi: 10.4049/jimmunol.1600126 PMC5360537 28228560

[82] Nograles KE Zaba LC Shemer A Fuentes-Duculan J Cardinale I Kikuchi T. Il-22-producing "T22" T cells account for upregulated il-22 in atopic dermatitis despite reduced il-17-producing th17 T cells. J Allergy Clin Immunol (2009) 123(6):1244–52.e2. doi: 10.1016/j.jaci.2009.03.041 19439349 PMC2874584

[83] Guttman-Yassky E Brunner PM Neumann AU Khattri S Pavel AB Malik K. Efficacy and safety of fezakinumab (an il-22 monoclonal antibody) in adults with moderate-to-severe atopic dermatitis inadequately controlled by conventional treatments: A randomized, double-blind, phase 2a trial. J Am Acad Dermatol (2018) 78(5):872–81.e6. doi: 10.1016/j.jaad.2018.01.016 29353025 PMC8711034

[84] Puya R Alvarez-López M Velez A Casas Asuncion E Moreno JC. Treatment of severe refractory adult atopic dermatitis with ustekinumab. Int J Dermatol (2012) 51(1):115–6. doi: 10.1111/j.1365-4632.2011.05195.x 22182388

[85] Lis-Święty A Skrzypek-Salamon A Arasiewicz H Brzezińska-Wcisło L. Atopic dermatitis exacerbated with ustekinumab in a psoriatic patient with childhood history of atopy. Allergol Int (2015) 64(4):382–3. doi: 10.1016/j.alit.2015.06.003 26433537

[86] Watt FM Estrach S Ambler CA. Epidermal notch signalling: differentiation, cancer and adhesion. Curr Opin Cell Biol (2008) 20(2):171–9. doi: 10.1016/j.ceb.2008.01.010 PMC2324124 18342499

[87] Gratton R Tricarico PM Moltrasio C Lima Estevão de Oliveira AS Brandão L Marzano AV. Pleiotropic role of notch signaling in human skin diseases. Int J Mol Sci (2020) 21(12):4214. doi: 10.3390/ijms21124214 32545758 PMC7353046

[88] Dumortier A Durham AD Di Piazza M Vauclair S Koch U Ferrand G. Atopic dermatitis-like disease and associated lethal myeloproliferative disorder arise from loss of notch signaling in the murine skin. PloS One (2010) 5(2):e9258. doi: 10.1371/journal.pone.0009258 20174635 PMC2823782

[89] Smith SH Jayawickreme C Rickard DJ Nicodeme E Bui T Simmons C. Tapinarof Is a Natural Ahr Agonist That resolves Skin Inflammation in Mice and humans. J Invest Dermatol (2017) 137(10):2110–9. doi: 10.1016/j.jid.2017.05.004 28595996

[90] Denison MS Nagy SR. Activation of the aryl hydrocarbon receptor by structurally diverse exogenous and endogenous chemicals. Annu Rev Pharmacol Toxicol (2003) 43:309–34. doi: 10.1146/annurev.pharmtox.43.100901.135828 12540743

[91] Stockinger B Di Meglio P Gialitakis M Duarte JH. The aryl hydrocarbon receptor: multitasking in the immune system. Annu Rev Immunol (2014) 32:403–32. doi: 10.1146/annurev-immunol-032713-120245 24655296

[92] Li S Heller JJ Bostick JW Lee A Schjerven H Kastner P. Ikaros inhibits group 3 innate lymphoid cell development and function by suppressing the aryl hydrocarbon receptor pathway. Immunity (2016) 45(1):185–97. doi: 10.1016/j.immuni.2016.06.027 PMC4959810 27438771

[93] Qiu J Guo X Chen ZM He L Sonnenberg GF Artis D. Group 3 innate lymphoid cells inhibit T-cell-mediated intestinal inflammation through aryl hydrocarbon receptor signaling and regulation of microflora. Immunity (2013) 39(2):386–99. doi: 10.1016/j.immuni.2013.08.002 PMC3884586 23954130

[94] Qiu J Heller JJ Guo X Chen ZM Fish K Fu YX. The aryl hydrocarbon receptor regulates gut immunity through modulation of innate lymphoid cells. Immunity (2012) 36(1):92–104. doi: 10.1016/j.immuni.2011.11.011 22177117 PMC3268875

[95] Li S Bostick JW Ye J Qiu J Zhang B Urban JF Jr.. Aryl hydrocarbon receptor signaling cell intrinsically inhibits intestinal group 2 innate lymphoid cell function. Immunity (2018) 49(5):915–28.e5. doi: 10.1016/j.immuni.2018.09.015 30446384 PMC6249058

[96] Craig JM. Atopic dermatitis and the intestinal microbiota in humans and dogs. Veterinary Med Sci (2016) 2(2):95–105. doi: 10.1002/vms3.24 PMC5645856 29067183

[97] Furue M Tsuji G Mitoma C Nakahara T Chiba T Morino-Koga S. Gene regulation of filaggrin and other skin barrier proteins via aryl hydrocarbon receptor. J Dermatol Sci (2015) 80(2):83–8. doi: 10.1016/j.jdermsci.2015.07.011 26276439

[98] Lee J Song KM Jung CH. Diosmin restores the skin barrier by targeting the aryl hydrocarbon receptor in atopic dermatitis. Phytomedicine (2021) 81:153418. doi: 10.1016/j.phymed.2020.153418 33302042

[99] van den Bogaard EH Bergboer JG Vonk-Bergers M van Vlijmen-Willems IM Hato SV van der Valk PG. Coal tar induces ahr-dependent skin barrier repair in atopic dermatitis. J Clin Invest (2013) 123(2):917–27. doi: 10.1172/jci65642 PMC3561798 23348739

[100] Paller AS Stein Gold L Soung J Tallman AM Rubenstein DS Gooderham M. Efficacy and patient-reported outcomes from a phase 2b, randomized clinical trial of tapinarof cream for the treatment of adolescents and adults with atopic dermatitis. J Am Acad Dermatol (2021) 84(3):632–8. doi: 10.1016/j.jaad.2020.05.135 32502588

[101] Peppers J Paller AS Maeda-Chubachi T Wu S Robbins K Gallagher K. A phase 2, randomized dose-finding study of tapinarof (Gsk2894512 cream) for the treatment of atopic dermatitis. J Am Acad Dermatol (2019) 80(1):89–98.e3. doi: 10.1016/j.jaad.2018.06.047 30554600

[102] Thammahong A Kiatsurayanon C Edwards SW Rerknimitr P Chiewchengchol D. The clinical significance of fungi in atopic dermatitis. Int J Dermatol (2020) 59(8):926–35. doi: 10.1111/ijd.14941 32441807

[103] Tata A Dodard G Fugère C Leget C Ors M Rossi B. Combination blockade of klrg1 and pd-1 promotes immune control of local and disseminated cancers. Oncoimmunology (2021) 10(1):1933808. doi: 10.1080/2162402x.2021.1933808 34188973 PMC8208121

[104] Borys SM Bag AK Brossay L Adeegbe DO. The yin and yang of targeting klrg1(+) tregs and effector cells. Front Immunol (2022) 13:894508. doi: 10.3389/fimmu.2022.894508 35572605 PMC9098823

[105] Van den Bossche J Van Ginderachter JA. E-cadherin: from epithelial glue to immunological regulator. Eur J Immunol (2013) 43(1):34–7. doi: 10.1002/eji.201243168 23229729

[106] Trautmann A Altznauer F Akdis M Simon HU Disch R Bröcker EB. The differential fate of cadherins during T-cell-induced keratinocyte apoptosis leads to spongiosis in eczematous dermatitis. J Invest Dermatol (2001) 117(4):927–34. doi: 10.1046/j.0022-202x.2001.01474.x 11676834

[107] Turner CT Zeglinski MR Richardson KC Santacruz S Hiroyasu S Wang C. Granzyme B contributes to barrier dysfunction in oxazolone-induced skin inflammation through E-cadherin and flg cleavage. J Invest Dermatol (2021) 141(1):36–47. doi: 10.1016/j.jid.2020.05.095 32504614

[108] Turner CT Lim D Granville DJ. Granzyme B in skin inflammation and disease. Matrix Biol (2019) 75-76:126–40. doi: 10.1016/j.matbio.2017.12.005 29247692

[109] Kamata Y Kimura U Matsuda H Tengara S Kamo A Umehara Y. Relationships among plasma granzyme B level, pruritus and dermatitis in patients with atopic dermatitis. J Dermatol Sci (2016) 84(3):266–71. doi: 10.1016/j.jdermsci.2016.09.009 27686401

[110] Honda T Kabashima K. Prostanoids in allergy. Allergol Int (2015) 64(1):11–6. doi: 10.1016/j.alit.2014.08.002 25572554

[111] Inagaki S Nakamura T Hamasaki Y Yamamoto-Hanada K Fukuie T Narita M. Prostaglandin D(2) metabolite is not a useful clinical indicator for assessing atopic dermatitis. Clin Exp Dermatol (2021) 46(1):130–4. doi: 10.1111/ced.14393 32705704

[112] Sarashina H Tsubosaka Y Omori K Aritake K Nakagawa T Hori M. Opposing immunomodulatory roles of prostaglandin D2 during the progression of skin inflammation. J Immunol (2014) 192(1):459–65. doi: 10.4049/jimmunol.1302080 24298012

[113] Maric J Ravindran A Mazzurana L Van Acker A Rao A Kokkinou E. Cytokine-induced endogenous production of prostaglandin D(2) is essential for human group 2 innate lymphoid cell activation. J Allergy Clin Immunol (2019) 143(6):2202–14.e5. doi: 10.1016/j.jaci.2018.10.069 30578872

[114] Xue L Salimi M Panse I Mjösberg JM McKenzie AN Spits H. Prostaglandin D2 activates group 2 innate lymphoid cells through chemoattractant receptor-homologous molecule expressed on th2 cells. J Allergy Clin Immunol (2014) 133(4):1184–94. doi: 10.1016/j.jaci.2013.10.056 PMC3979107 24388011

[115] Wojno ED Monticelli LA Tran SV Alenghat T Osborne LC Thome JJ. The prostaglandin D_2_ Receptor crth2 regulates accumulation of group 2 innate lymphoid cells in the inflamed lung. Mucosal Immunol (2015) 8(6):1313–23. doi: 10.1038/mi.2015.21 PMC4598246 25850654

[116] Heise CE O'Dowd BF Figueroa DJ Sawyer N Nguyen T Im DS. Characterization of the human cysteinyl leukotriene 2 receptor. J Biol Chem (2000) 275(39):30531–6. doi: 10.1074/jbc.M003490200 10851239

[117] Doherty TA Khorram N Lund S Mehta AK Croft M Broide DH. Lung type 2 innate lymphoid cells express cysteinyl leukotriene receptor 1, which regulates th2 cytokine production. J Allergy Clin Immunol (2013) 132(1):205–13. doi: 10.1016/j.jaci.2013.03.048 PMC3704056 23688412

[118] Salimi M Stöger L Liu W Go S Pavord I Klenerman P. Cysteinyl leukotriene E(4) activates human group 2 innate lymphoid cells and enhances the effect of prostaglandin D(2) and epithelial cytokines. J Allergy Clin Immunol (2017) 140(4):1090–100.e11. doi: 10.1016/j.jaci.2016.12.958 28115217 PMC5624780

[119] Golub R. The notch signaling pathway involvement in innate lymphoid cell biology. BioMed J (2021) 44(2):133–43. doi: 10.1016/j.bj.2020.12.004 PMC8178581 33863682

[120] Yu X Vargas J Green PHR Bhagat G. Innate lymphoid cells and celiac disease: current perspective. Cell Mol Gastroenterol Hepatol (2021) 11(3):803–14. doi: 10.1016/j.jcmgh.2020.12.002 PMC7851184 33309944

